# Minimally invasive management of deep incisional infection following posterior spinal surgery: a case report

**DOI:** 10.3389/fmed.2025.1606535

**Published:** 2025-07-21

**Authors:** Hongbin Wang, Jing Xu, Lanlan Pang, Luyue Bai, Xutao Fan

**Affiliations:** ^1^Jining Medical University, Jining, China; ^2^Department of Infectious Diseases, Affiliated Hospital of Jining Medical University, Jining, China; ^3^Department of Medical Ultrasonics, Affiliated Hospital of Jining Medical University, Jining, China; ^4^Spine Surgery, Affiliated Hospital of Jining Medical University, Jining, Chin

**Keywords:** posterior cervical surgery, postoperative infection, ultrasound-guided, catheter drainage, minimally invasive therapy

## Abstract

**Background:**

In recent years, innovations in spinal surgical techniques have significantly improved clinical outcomes and quality of life for patients with spinal diseases. However, the risk of deep surgical site infection (SSI) following posterior approaches remains a significant concern. Through analysis of a representative case, this study systematically explores the clinical value of ultrasound-guided percutaneous catheter drainage (PCD) in managing deep incisional infections following posterior spinal surgery, this approach offers an innovative solution for postoperative deep infections.

**Methods:**

A patient with cervical hyperextension injury caused by drowning underwent posterior cervical expansive open-door laminoplasty with internal fixation. Postoperative deep incisional infection occurred and was treated with ultrasound-guided PCD combined with systemic antibiotic therapy. The infected cavity was irrigated twice daily with saline and maintained under continuous negative-pressure suction. Infection-related biomarkers and abscess size were closely monitored to evaluate the efficacy of this approach.

**Results:**

The patient exhibited symptomatic relief, significant reduction in abscess volume, and normalization of inflammatory markers, successfully avoiding secondary open debridement. This approach minimized surgical trauma and nursing challenges. A 12-month follow-up confirmed no infection recurrence and maintained cervical stability.

**Conclusion:**

Ultrasound-guided percutaneous drainage, with its advantages of minimal invasiveness, safety, and real-time visualization, represents a promising first-line therapeutic option for deep incisional infections following posterior spinal surgery.

## 1 Introduction

Surgical site infection (SSI) affects 3.1% of spinal surgeries, with deep incisional infections posing significant challenges due to prolonged healing and risks of implant removal or spinal instability (1). Deep incisional SSI following spinal procedures represents a severe complication requiring comprehensive diagnostic evaluation through clinical manifestations, physical signs, and laboratory/imaging investigations. Localized tenderness, edema, and erythema at the surgical site constitute the most frequent clinical presentations of deep infection after posterior spinal approaches (2). In immunocompromised patients, delayed intervention may lead to life-threatening sequelae including sepsis, end-organ failure, and mortality (3). Early therapeutic intervention is therefore imperative upon confirmation of diagnosis in clinical practice.

Traditional treatments, such as open debridement and vacuum-assisted closure (VAC), often require repeated procedures under general anesthesia, increasing complications and delaying rehabilitation (4). Recent advances in image-guided techniques provide alternatives for minimizing invasiveness. This report highlights the clinical efficacy of ultrasound-guided percutaneous catheter drainage in managing deep cervical postoperative infections.

Our hospital recently encountered a representative case involving a patient who developed a deep incisional infection after undergoing posterior cervical expansive open-door laminoplasty with internal fixation for a cervical hyperextension injury caused by drowning. Considering the significant trauma associated with traditional debridement strategies which not only prolong hospitalization but also increase therapeutic and nursing complexities, impose additional financial burdens on patients, and adversely affect rehabilitation outcomes we adopted a minimally invasive approach combining ultrasound-guided PCD with antibiotic therapy. This case report demonstrates the clinical value of ultrasound-guided PCD in the management of such infections.

## 2 Case presentation

A 60-year-old male presented with limb weakness after drowning-related cervical hyperextension injury. Preoperative MRI revealed multilevel disc herniation and spinal stenosis. Posterior cervical laminoplasty (C3–C7) with internal fixation was performed. Postoperatively, the patient regained ambulatory capacity with a cervical brace. The patient was usually in good health, with a surgical history of “appendectomy” 10 years ago, and no family history of hereditary diseases.

**Figure 1:**
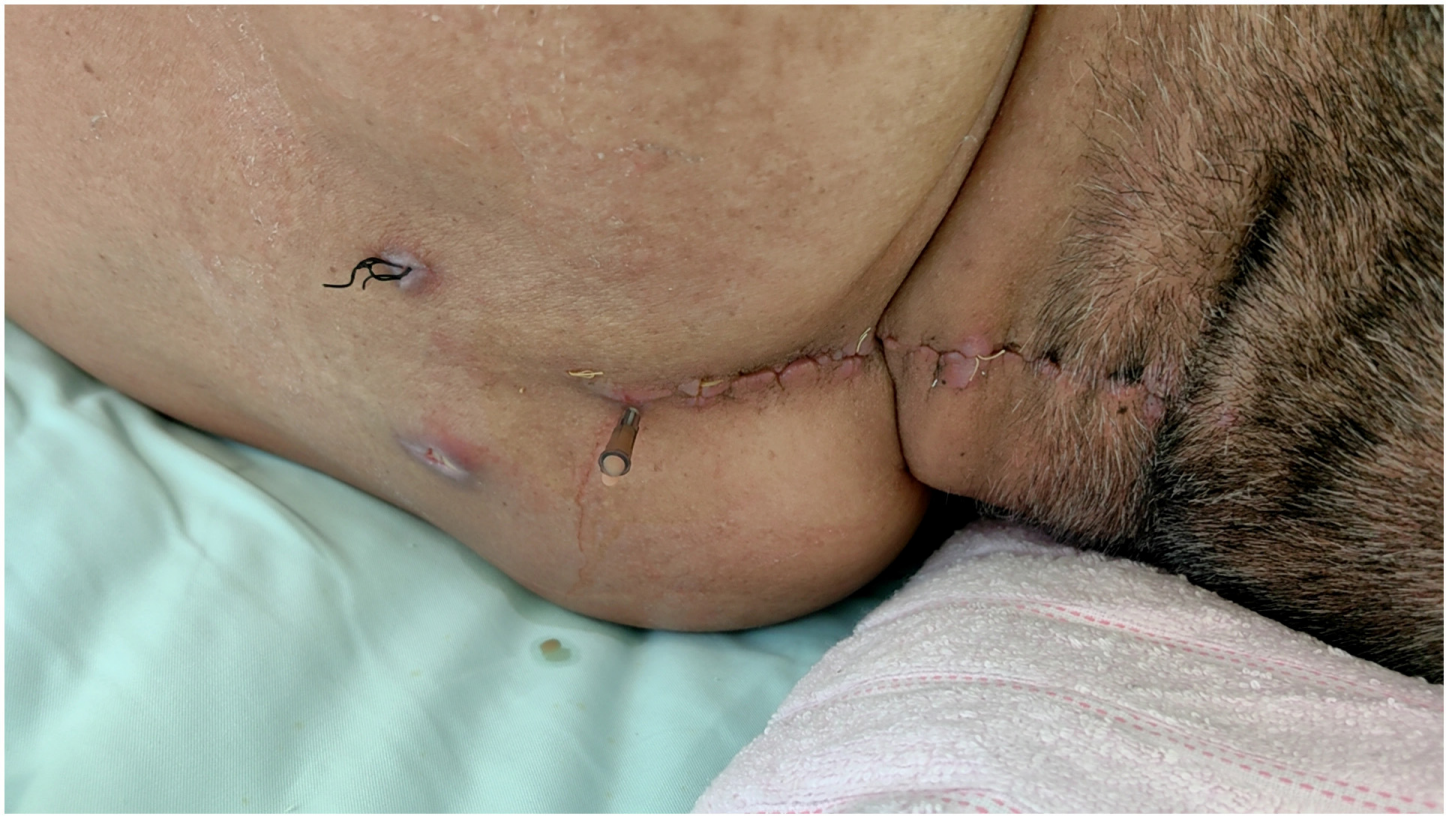
On postoperative day 15, the cervical incision exhibits mild erythema and swelling without purulent discharge. A small amount of serous fluid is observed exuding from the original surgical drain site.

**Figure 2:**
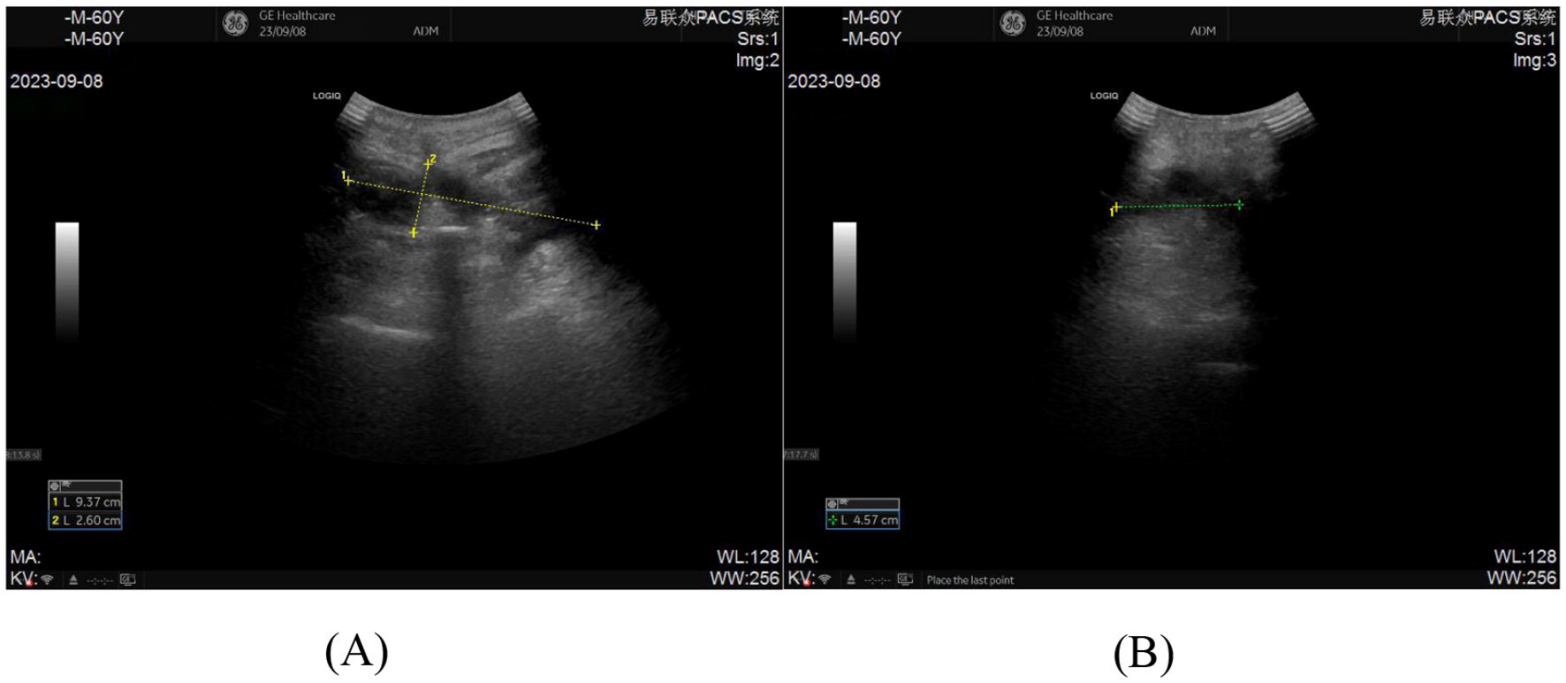
**(A, B)** Pre-procedural ultrasonographic imaging of the incision for ultrasound-guided percutaneous catheter drainage demonstrates an abscess measuring 9.37 cm × 2.60 cm × 4.57 cm.

On postoperative day 12, the patient developed fever with occasional cough. A dressing change revealed no signs of erythema, swelling, heat, or pain at the incision site, which exhibited satisfactory healing. Laboratory tests showed leukocytosis (14.21 × 10^9^/L) and elevated C-reactive protein (104.22 mg/L). The symptoms were initially attributed to aspiration pneumonia secondary to drowning. Prompted by this suspicion, cefathiamidine antibiotic therapy was initiated. However, after 3 days of treatment, the patient remained febrile with persistent cough and developed cervicodorsal pain. Repeat laboratory tests demonstrated decreased leukocyte count (10.42 × 10^9^/L) but further elevated inflammatory markers (C-reactive protein: 205.31 mg/L; erythrocyte sedimentation rate: 82.00 mm/h). Suspecting surgical site infection, the incision was re-evaluated, revealing minimal serous discharge at the distal drainage orifice (Figure 1), and subsequent ultrasonography identified a deep encapsulated fluid collection (9.37 cm × 2.60 cm × 4.57 cm) within the surgical field, confirming the diagnosis of postoperative infection (Figures 2A, B).

**Figure 3:**
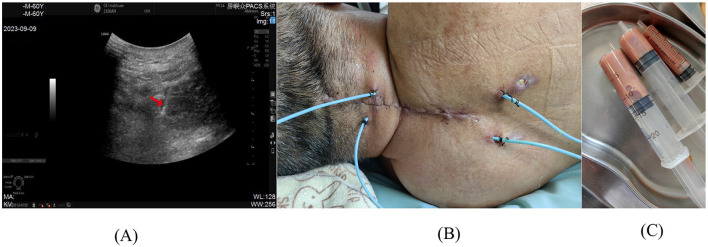
**(A)** Ultrasonographic needle trajectory during catheter placement, with arrow indicating the puncture needle; **(B)** External segment of the drainage catheter post-deployment; **(C)** Coffee-colored purulent fluid aspirated through the catheter following successful placement.

Following multidisciplinary team (MDT) consensus, an ultrasound-guided percutaneous catheter drainage protocol was formulated. Four drainage tubes (Commercial name: DIALL Dial; guidewire-introduced straight type: 7Fr × 2 for irrigation, 10Fr × 2 for drainage).were inserted under real-time sonographic guidance adjacent to the surgical incision: Two 10Fr tubes served as drainage conduits under continuous negative pressure (Negative pressure during irrigation was 6 kPa; during non-irrigation periods, connected to a negative pressure drainage bulb for continuous drainage of exudate, etc.), and two 7Fr tubes were dedicated to twice-daily saline irrigation with 100 mL saline to cleanse the abscess cavity (Figures 3A, B). This was complemented by intravenous vancomycin (1 g, q12h) therapy. After successful catheter placement, ~25 ml of coffee-ground purulent fluid was aspirated with injectate to confirm drain patency (Figure 3C).

**Figure 4:**
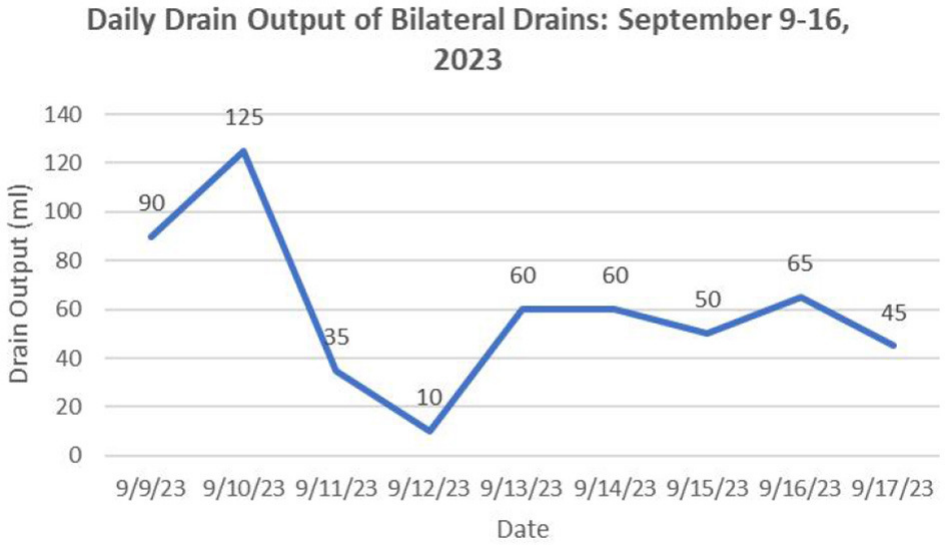
During the period from successful catheter insertion until removal, the daily drainage volume (total volume from both catheters).

**Figure 5:**
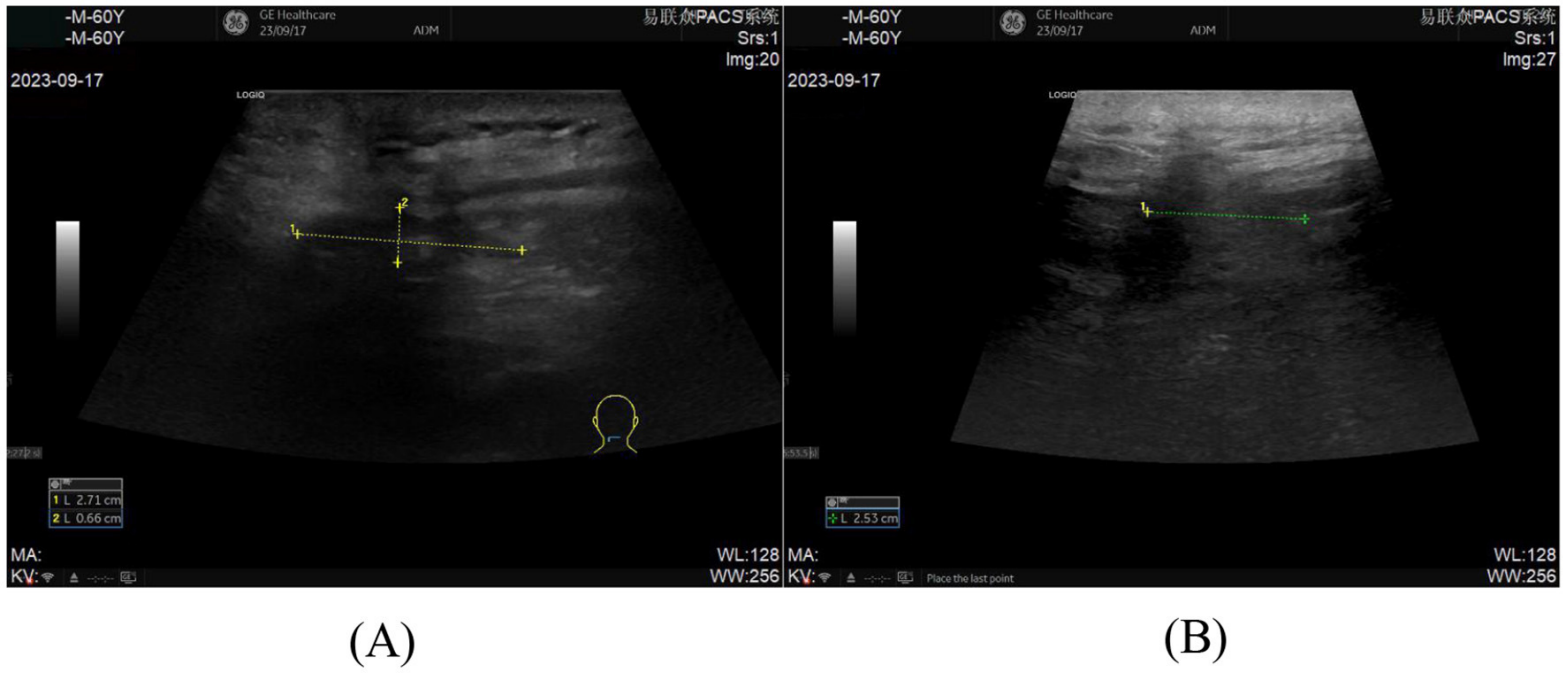
**(A, B)** Follow-up ultrasonography on postoperative day 7 after ultrasound guided catheter drainage demonstrates a residual anechoic fluid collection measuring approximately 2.71 cm × 0.66 cm × 2.53 cm.

Microbiological culture obtained on post-catheterization day 3 identified *Enterobacter cloacae* infection, prompting antibiotic adjustment to moxifloxacin hydrochloride based on antimicrobial susceptibility testing. Clinical improvement was evidenced by resolution of fever within 24 h post-procedure and progressive normalization of drainage output, with serous yellow effluent (< 50 mL/day) observed by day 7 (Figure 4). Inflammatory markers demonstrated significant reduction: leukocyte count 7.14 × 10^9^/L, CRP 18.93 mg/L, ESR 31.00 mm/h, and procalcitonin 0.042 ng/mL (all ≤ 2 × upper limit of normal), alongside sustained apyrexia for 15 consecutive days. Follow-up ultrasound revealed the anechoic area (2.71 cm × 0.66 cm × 2.53 cm) was reduced by 95.94% compared to pre-drainage (Figures 5A, B), prompting catheter removal following multidisciplinary review.

Post-removal hospitalization included 7 days of continued antimicrobial therapy, during which the incision exhibited primary healing without signs of inflammation (erythema/swelling/purulence). Repeat biomarkers confirmed inflammatory resolution: leukocytes 7.70 × 10^9^/L, CRP 6.53 mg/L, ESR 9.00 mm/h, and procalcitonin 0.036 ng/mL. The patient was discharged with a 4-week oral moxifloxacin regimen and scheduled biweekly outpatient evaluations. Two consecutive follow-ups demonstrated absence of abscess recurrence on imaging and normalization of all biomarkers (leukocytes, CRP, ESR, procalcitonin), meeting predefined criteria for antimicrobial cessation. During 12-month surveillance, the patient maintained infection-free status with preserved cervical spine stability, as confirmed by dynamic.

During hospitalization, the patient expressed: due to drowning, was psychologically unable to endure reoperation, was worried that the condition could not be cured, and would affect family life, experienced profound anxiety about the future. However, after hearing our minimally invasive treatment plan, gained motivation to persist with treatment. After a period of treatment, seeing the condition improve, the patient gradually regained confidence, mentality became optimistic and positive; post-recovery, the patient expressed cherishing health more, having new insights about life.

## 3 Discussion

This study describes a minimally invasive approach for managing deep incisional infections following posterior spinal surgery. Our protocol employed ultrasound-guided percutaneous catheterization with continuous drainage combined with systemic antibiotic therapy. Postoperative management involved twice-daily saline irrigation of the abscess cavity coupled with continuous negative-pressure suction, resulting in effective control of infection-related parameters and progressive reduction in abscess cavity dimensions. The implemented strategy ultimately led to successful patient recovery and discharge.

Clinical evidence identifies multiple risk factors for postoperative spinal infections, including advanced age, obesity, diabetes mellitus, smoking history, prior surgical interventions, surgical approaches, spinal instrumentation, and intraoperative environmental parameters (5, 6). Notably, Pennington et al. (7) demonstrated that postoperative hyperglycemia and suboptimal glycemic control constitute independent risk factors for surgical site infections in spinal procedures. Comparative studies further suggest increased infection susceptibility in patients with advanced age, multi-level instrumentation, substantial intraoperative blood loss, elevated transfusion requirements, and prolonged operative trauma (8). In the present case, the patient's prior drowning episode represented a significant infection risk determinant. Although standard perioperative antibiotic prophylaxis was administered following initial surgery, subsequent development of *Enterobacter cloacae* infection confirmed by microbial culture revealed a strong epidemiological association with water exposure history.

Current clinical practice lacks standardized protocols for managing post-instrumentation spinal infections. Deep incisional surgical site infection (DSSI) is conventionally managed with debridement combined with vacuum-assisted closure (VAC) therapy under continuous negative pressure (9). However, this method has significant limitations and disadvantages: First is its strong invasiveness, requiring thorough wound opening and debridement to be performed in the operating room, causing greater trauma. Second is cumbersome procedures and prolonged healing cycles, requiring waiting for adequate granulation tissue formation before secondary suture closure can be performed, extending the recovery time (10). Moreover, each VSD dressing change must be done under sterile conditions in the operating room, making the process complex. Third, there exist risks of repeated surgical operations, anesthesia exposure, and potential poor wound healing.

In contrast, ultrasound-guided percutaneous catheter drainage offers distinct advantages: First, the minimally invasive procedure avoids the greater trauma of open surgery, achieved only through percutaneous catheter placement. Second, it is accurate, safe, convenient, and efficient: real-time ultrasound guidance clearly displays important vascular and neural structures, significantly reducing the risk of accidental injury. Moreover, the catheter placement operation is relatively simple and can be performed under non-general anesthesia; postoperative irrigation and drainage management can be completed in the ward, eliminating repeated visits to the operating room for dressing changes. Third, patient care and rehabilitation improvement: this technique preserves patient mobility, reducing risks of deep vein thrombosis, hypostatic pneumonia, and psychological distress associated with prolonged bed rest. Enhanced patient compliance improves nutritional intake and accelerates rehabilitation. Fourth, continuous irrigation effectively dilutes necrotic debris, disrupts bacterial colonization by eliminating nutrient substrates, maintains low bacterial bioburden, optimizes wound microenvironments, and enhances microbial clearance rates—collectively suppressing inflammatory cascades and preventing cross-infection (11, 12).

However, contraindications include cerebrospinal fluid leaks or active hemorrhage, where negative pressure may exacerbate fluid loss or hemorrhage, and anaerobic infections exacerbated by closed drainage systems (13). Ideal candidates exhibit well-demarcated encapsulated infections without superficial wound compromise, cerebrospinal fluid leakage, or neurological compression. The technique is unsuitable for deep infections with wound dehiscence, multiloculated abscesses, viscous purulent collections, or compressive neurological pathologies.

## 4 Conclusion

Ultrasound-guided percutaneous catheter drainage is a safe, effective, and minimally invasive strategy for deep cervical postoperative infections. Combined with multidisciplinary care, it optimizes patient outcomes and reduces the need for revision surgery.

## Data Availability

The original contributions presented in the study are included in the article/supplementary material, further inquiries can be directed to the corresponding author.
